# High Resolution Crystal Structure of Human β-Glucuronidase Reveals Structural Basis of Lysosome Targeting

**DOI:** 10.1371/journal.pone.0079687

**Published:** 2013-11-19

**Authors:** Md. Imtaiyaz Hassan, Abdul Waheed, Jeffery H. Grubb, Herbert E. Klei, Sergey Korolev, William S. Sly

**Affiliations:** 1 Edward A. Doisy Department of Biochemistry and Molecular Biology, Saint Louis University School of Medicine, Saint Louis, Missouri, United States of America; 2 Centre for Interdisciplinary Research in Basic Sciences, Jamia Millia Islamia, Jamia Nagar, New Delhi, India; 3 Research and Development, Bristol-Myers Squibb Company, Princeton, New Jersey, United States of America; Griffith University, Australia

## Abstract

Human β-glucuronidase (GUS) cleaves β-D-glucuronic acid residues from the non-reducing termini of glycosaminoglycan and its deficiency leads to mucopolysaccharidosis type VII (MPSVII). Here we report a high resolution crystal structure of human GUS at 1.7 Å resolution and present an extensive analysis of the structural features, unifying recent findings in the field of lysosome targeting and glycosyl hydrolases. The structure revealed several new details including a new glycan chain at Asn272, in addition to that previously observed at Asn173, and coordination of the glycan chain at Asn173 with Lys197 of the lysosomal targeting motif which is essential for phosphotransferase recognition. Analysis of the high resolution structure not only provided new insights into the structural basis for lysosomal targeting but showed significant differences between human GUS, which is medically important in its own right, and *E. coli* GUS, which can be selectively inhibited in the human gut to prevent prodrug activation and is also widely used as a reporter gene by plant biologists. Despite these differences, both human and *E. coli* GUS share a high structure homology in all three domains with most of the glycosyl hydrolases, suggesting that they all evolved from a common ancestral gene.

## Introduction

Human β-glucuronidase (GUS) acts as an exoglycosidase in lysosomes and is involved in stepwise degradation of glucuronic acid-containing glycosaminoglycans (GAGs) including heparan sulfate, dermatan sulfate, and chondroitin sulfate [1], [2]. The functional form of GUS is a tetramer of four identical subunits of 75000 Da [3]. It is a member of the family of β-glycosidases (Family 2) that includes β-glucuronidase, β-galactosidase, and β-mannosidase [4], [5], [6]. The gene encoding human GUS is present on chromosome 7 [7], [8]. Sequence analysis indicates there are four potential glycosylation sites and biochemical analysis indicates that all are glycosylated [9], [10]. This enzyme is of great importance because it hydrolyzes GAGs, and its deficiency causes mucopolysaccharidosis type VII (MPSVII) [11], also known as Sly syndrome [12]. In the absence of GUS, chondroitin sulfate, dermatan sulfate, and heparan sulfate are only partially degraded and accumulate in the lysosomes of many tissues. This enzyme is widely used as a therapeutic molecule for experimental enzyme replacement therapy in animal models of MPSVII [13], [14].

The transport of enzymes from their site of synthesis (rough endoplasmic reticulum) to lysosomes is mediated by a series of protein and carbohydrate recognition signals present on the sequence or structure of the enzyme [15], [16], [17]. Transport depends on the degree of glycosylation and recognition of glycosylated sites by phosphotransferase [18], [19]. Phosphorylation of mannose residues on N-linked oligosaccharide side chains of lysosomal enzymes targets them to lysosomes. The phosphorylation of terminal mannose residues is completed in two distinct steps: transfer of N-acetylglucosamine-1-phosphate (GlcNAc-1-P) from uridine diphosphate N-acetylglucosamine (UDP-GlcNAc) to the 6-position of mannose residues on high mannose-type oligosaccharide chains by the enzyme UDP-GlcNAc phosphotransferase and the removal of covering GlcNAc residues to generate phospho- monoesters of mannose by the enzyme N-acetylglucosaminyl phosphodiesterase [20], [21], [22]. The phosphotransferase recognizes a unique conformation signal shared by lysosomal enzymes that is not present in other secretory proteins [23]. Subsequently, the mannose-6-phosphate residues are recognized in the trans-Golgi network by specific receptors that transport lysosomal enzymes to lysosomes [24].

The three-dimensional structure of human GUS was previously reported at 2.6 Å resolution [25]. The structure of the monomer contains three distinct domains: Jelly roll barrel (residues 22–223), an immunoglobulin region constant domain (residues 224–342), and a TIM barrel domain (residues 342–632) [25]. Site-directed mutagenesis studies showed that Glu451, Glu540, and Tyr504 play essential roles in catalysis [26]. One amino acid acts as a catalytic nucleophile (Glu540) and the other as an acid-base catalyst or the proton donor (Glu451) [1]. The jelly roll barrel domain contains important residues for lysosomal targeting. Structural and biochemical studies on cathepsin D, suggested that lysosomal enzyme recognition motifs include Lys203 and the loop formed by residues 265–293 of cathepsin D [27]. These residues correspond to Lys197 and residues 179–201 of human GUS [25], [27]. Mutagenesis studies of several lysosomal enzymes such as DNase I [28], aspartylglucosaminidase (AGA) [29], and aryl sulfatase [30] show that each contains surface lysines as essential components of their phosphotransferase recognition domain.

Genetic deficiency of human GUS enzyme leads to accumulation of undegraded GAGs in lysosomes and produces the clinical disorder called Sly syndrome [31]. Studies of human GUS led to the discovery of the mannose 6-phosphate (Man6P) recognition marker, which targets acid hydrolases to lysosomes, and helped define the Man6P receptor-dependent pathways for delivery of acid hydrolases to lysosomes [32], [33]. A large number of reports have focused on the biochemical studies of lysosomal targeting of GUS [14], [19], [21], [27] but only a single structure at 2.6 Å resolution has been published [25]. Higher resolution structural data for human GUS could provide additional insights into the lysosomal targeting mechanism of this enzyme. To obtain a high resolution crystal structure of human GUS we purified human GUS and produced a high quality crystal, which diffracted up to 1.7 Å resolution. Our analysis of the high resolution structure provides new insight for better understanding of the function of GUS and lysosomal targeting.

## Results and Discussion

### Sequence Analysis

**Figure 1:**
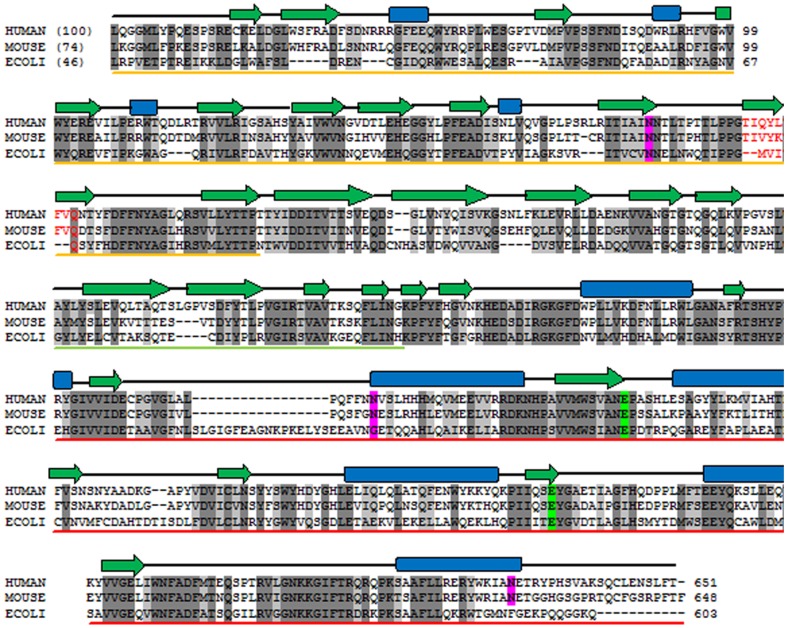
Multiple sequence alignment of human GUS with mouse and bacterial GUS. The percent sequence identities are given in parentheses. Completely conserved residues and homologous residues are shaded in dark and light grey, respectively. The secondary structure elements are given on the top of sequences, where α-helices are represented by blue rectangles, β-strands by green arrows. Domains 1, 2 and 3 are indicated by yellow, green and red line respectively, below the sequence. Conserved active site residues are highlighted in green boxes. Potential glycosylation sites are in pink. Glycosylation sites are in magenta boxes. Amino acid sequences of GUS were taken from the Uniprot database with their primary accession number as: human, P08236; mouse P12265; and *E. coli*, P05804.

The GUS polypeptide contains 629 residues and a 22 residue long signal sequence [5]. The sequence alignment of human GUS with other mammalian GUSs [34], [35], [36], [37] and *E. coli* GUS [38] is shown in **Fig. 1**, which indicates that sequences are highly conserved. Human GUS contains four potential glycosylation sites at Asn173, Asn272, Asn402 and Asn631 [10]. Sequence alignment reveals that Asn173 is completely conserved in all GUS. Two others, Asn402 and Asn631 are conserved only in mammalian sequences and are not present on *E. coli*. Asn272 is unique to the human sequence only.

### Structure Determination

The crystal structure of human GUS has been determined by the molecular replacement method and has been refined against diffraction data up to 1.7 Å resolution (**Table 1**). The crystals belong to the space group *P*2_1_2_1_2, with unit cell dimension a = 93. 58, b = 123.11, c = 266.14 and α = β = γ = 90°. Interestingly, the c axis is twice as large as the unit cell of the previously reported crystals [25], resulting in a tetramer occupying unit cell. The final R-factors for the structures are 20.7% (R_free_ = 24.2%). Root mean square deviations from ideal geometry are 0.018 Å and 1.90° for the respective bond lengths and angles. There are four monomers in the asymmetric unit and 70% solvent content in the cell. The final model contains residues 22 to 631 of each independent subunit, 2971 water molecules, six molecules of 2-methyl-2,4-pentanediol (MPD) and two sodium ions. The model has good stereochemistry and 99% of the residues fall within the allowed regions of the Ramachandran plot [39]. The *r.m.s*. differences between coordinates of C^α^ after superposition of the four subunits was lower than 0.2 Å and, therefore, all of the following discussion will be based on a single monomer.

**Table 1:** Crystallographic data and refinement statistics of human GUS.

pdb code	3HN3
Buffer/salt	10mM TrisHCl pH 7.5
Precipitating agent	30% MPD
**Data collection**:	
Wavelength (Å)	0.97
Space group	P 21 2121
Unit cell dimensions (Å)	a = 93.6
	b = 123.1
	c = 266.1
Molecules/asymmetric unit	4
Resolution range (Å)	20.0 – 1.67
Unique observations	316019
Completeness (%)^a^	88.4 (73.6)
R_sym_ (%)^a^	10.3 (48.8)
I/s(I)^a^	11.2 (1.2)
**Refinement**	
Resolution (Å)	20–1.7
R_cryst_ a	0.21 (0.26)
R_free_ a	0.24 (0.32)
Reflections (working/test)	276594/15611
Protein atoms	20011
No sugar atoms	572
Solvent molecules	2988
Rmsd bond lengths(Å)	0.009
Rmsd angles(Å^2^)	1.3
Rmsd DB (Å^2^) (mm/ms/ss)^b^	0.85/0.82/1.64
<B> protein (Å^2^)	14.7
<B> solvent (Å^2^)	26.1
**Ramachandran plot**:	
Most favored(%)	99.1
Generously allowed (%)	0.6
Disallowed (%)	0.3

a – numbers in parenthesis correspond to the highest resolution shell of (1.67–1.73 Å for data collection, 1.7–1.74 Å for refinement).

### Tertiary Structure

**Figure 2:**
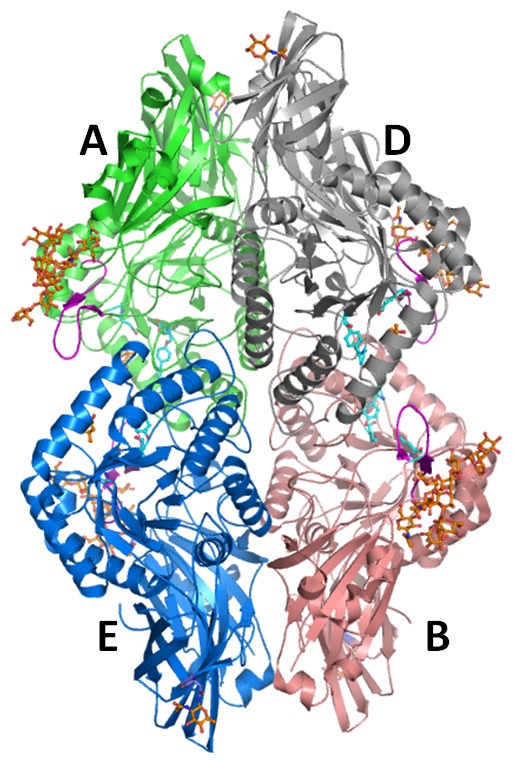
Overall structure of human GUS illustrated in cartoon model. Subunits A, B, D, and E are colored in green, light red, grey and sky blue, respectively. Residues involved in the catalysis are shown in ball and stick model (cyan) on each monomer. N-linked oligosaccharide chains are shown in ball and stick model (orange). The hairpin loop of each monomer is shown in magenta. All structures are drawn using the molecular visualization tool, PyMOL (The PyMOL Molecular Graphics System, Version 1.3, Schrödinger, LLC).

The overall structure of GUS contains four chains held together by non-covalent interactions (**Fig. 2**). The overall structural features deduced from the 1.7 Å data are similar to those of the reported structure at 2.6 Å resolution [25]. The electron density for glycan chains is observed at two positions in each monomer. A strong electron density was found between Asp362 of adjacent monomers (A–D and B–E) was surrounded by four additional water molecules, and were modeled as sodium ions. The independent active sites are located close to interfaces between A–E and B–D. The large interface areas of 1787 Å^2^ between A–D and B–E and 1944.3 Å^2^ between A–E and B–D corresponds to a stable oligomeric structural organization. This observation is consistent with the earlier report that GUS is functionally active as a dimer or tetramer but not as a monomer [40]. Furthermore, it supports the suggested lysosomal targeting motif of each monomer localizes near the same interface in the active site vicinity.

**Figure 3:**
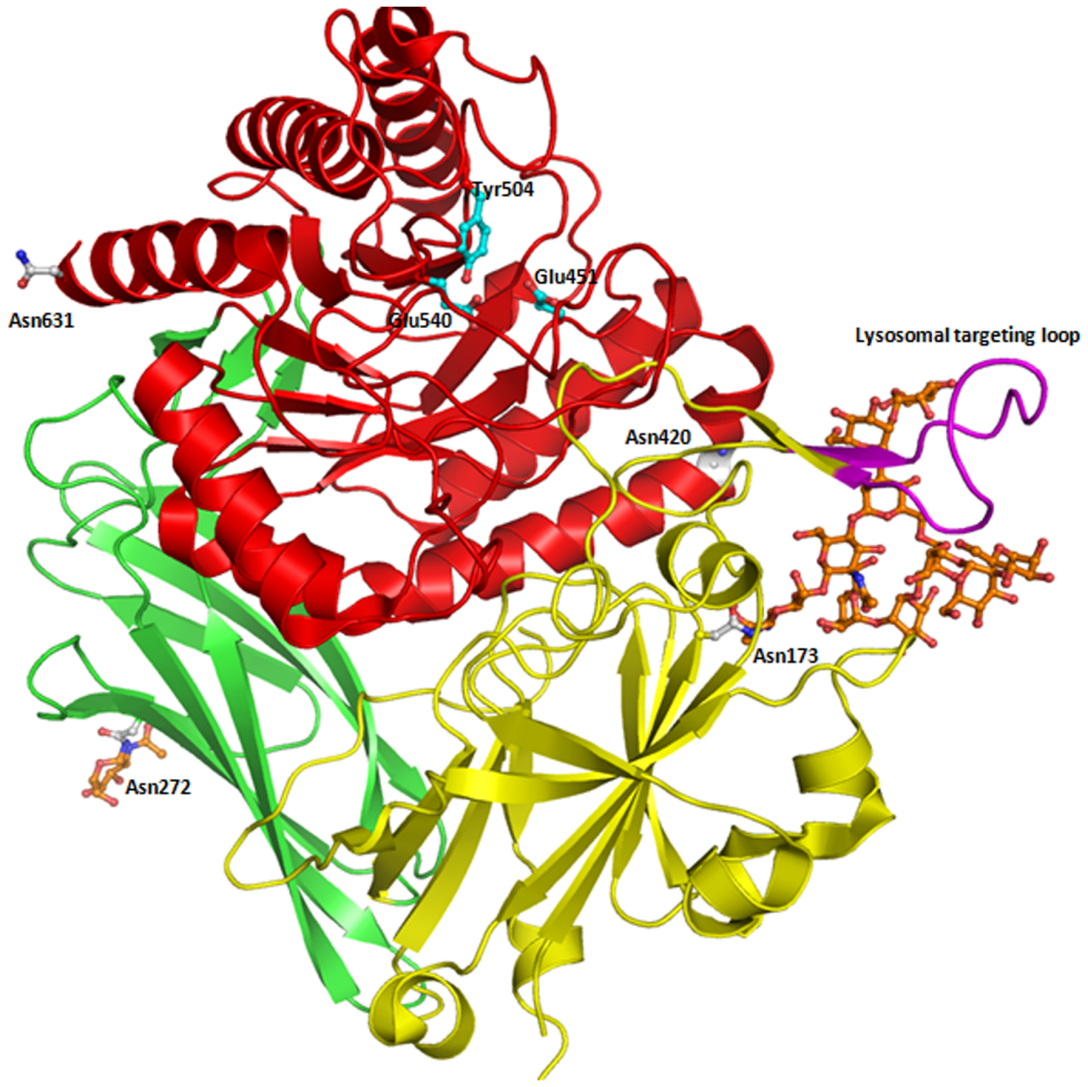
Structure of GUS monomer shown in cartoon model. Jelly roll domain, immunoglobulin constant region domain and TIM barrel domains are shown in yellow, green and red respectively. Residues involved in catalysis are shown in ball and stick model (cyan). Potential glycosylation sites and N-linked oligosaccharide chains are shown in light grey and orange respectively (ball and stick model). The hairpin loop proposed to be involved in lysosomal targeting is shown in pink.

The structure of each monomer was described in a previous publication [25] with three distinct domains (**Fig. 3**): a jelly roll barrel domain (22–223), an immunoglobulin constant region domain (224–242), and a TIM barrel domain (343–632). The loop comprising residues 189 to 199 is thought to be an important and common feature of mammalian lysosomal enzymes. Based on the sequence similarity and structural comparison with cathepsin D and arylsulfatase B, this loop is involved in phosphotransferase recognition [41], [42]. This loop forms a β-hairpin motif that is exposed on the surface and enables lysosomal enzymes recognition by phosphotransferase.

### Glycosylation

**Figure 4:**
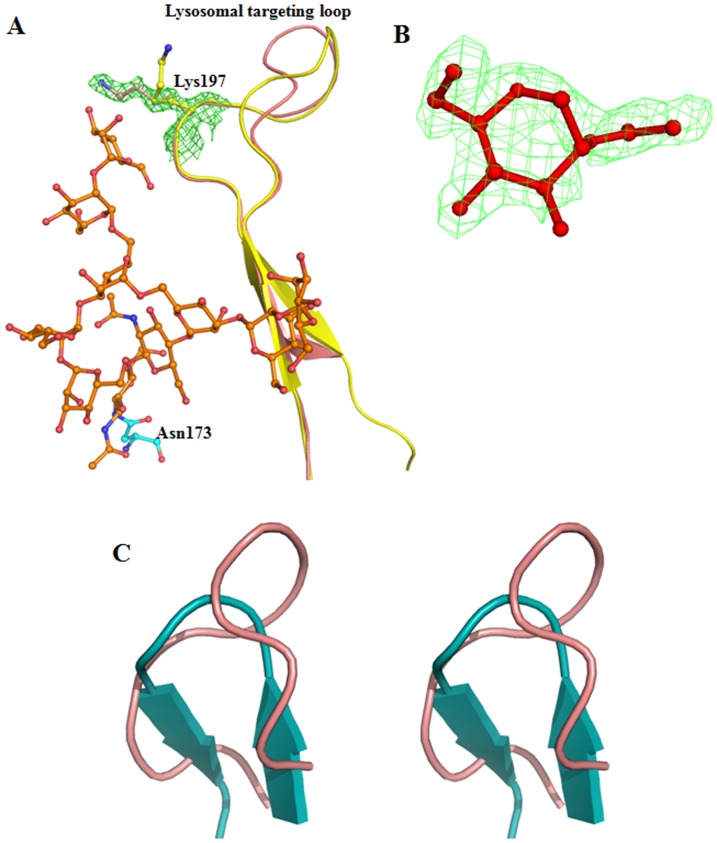
Representation of N-linked oligosaccharide chain on GUSB. (**A**). N-linked oligosaccharide chain at Asn173 and the lysosome targeting loop. Superimposed structure of reported earlier (1BHG, yellow) and the current structure (orange) of human GUS showing different orientation of lysosomal targeting motif. The side chain of Lys197, which is believed to participate in phosphotransferase recognition, is coordinated by the glycan chain at Asn173. **B**. N-linked oligosaccharide chain at Asn272. Contour electron density map (at the electron level 1.00 for a 2Fo-F) is shown with the modeled glycan chain. **C**. Stereo view of cartoon diagram showing a comparison of lysosomal target motif of human GUS (light red) with cathepsin D (cyan). The structure was drawn from the atomic coordinates of cathepsin D with pdb code, 1LYA [40].

The phosphorylation of mannose residues on the side chains of N-linked oligosaccharides of acid hydrolases like the GUS enzymes mediates their intracellular transport to lysosome and endocytes because the Man6P moieties are ligand for Man6P receptor [43]. In the case of cathepsin D, it was proposed that N-linked oligosaccharide chains at Asn70 and Asn199 plays key role in mannose 6P mediated lysosomal targeting [18], [41]. The sequence of human GUS contains four potential glycosylation sites (**Fig. 1**). Out of four glycosylation sites, two (Asn173 and Asn272) have a clear electron density that correspond to oligosaccharide chains in the crystal structure refined at 1.7 Å resolution. A hybrid N-linked oligosaccharide at Asn173 contains six mannose, one β-D-mannose and three N-acetyl glucosamine chains (**Fig. 4A**). An additional electron density was also observed corresponding to an extra terminal mannose residue not seen in the earlier structure. Importantly, the high-resolution structure revealed glycosylation of a second residue, Asn272, which was not previously observed. Asn272 contains a single N-acetyl-glucopyranose residue (**Fig. 4B**). Recently, site-specific glycoproteomic analysis revealed that Man7GlcNAc2-M6P oligosaccharides were present at Asn272 and Asn420, while Asn631 displayed Man6GlcNAc2-M6P [9]. We were unable to observe any electron density at Asn420 and Asn631. However, the presence of an appreciable electron density at Asn272 supports the role of this glycan chain in lysosomal targeting. In fact, earlier site directed mutagenesis studies of human GUS suggested that Asn272 and Asn420 were preferentially phosphorylated [10]. Elimination of these asparagines in combination markedly decreased sorting to lysosomes and increased enzyme secretion.

The glycan chain at Asn173 forms close interactions with multiple side chains including Arg56, Val96, Trp98, Tyr129, Thr175, Thr177, Thr185, Ile186, Gln187, Tyr188, Gly198 and Gln416. On the other hand, the glycan at Asn272 forms non-covalent interactions with Gly273 and Thr274. It has been reported that glycosylation is required for the formation of active enzyme, but that oligosaccharides can be removed enzymatically without significant loss of activity once the enzyme has folded properly [10]. The interactions of glycan chains to protein atoms clearly indicate a possible role in protein folding and stabilization. Mutation of different combinations of glycosylation sites led to reduction in enzyme activity, possibly because unglycosylated protein is unable to form soluble homotetramers [10]. Interestingly, Asn631 is present in the interface between monomers in the tetramer. Thus, it can be glycosylated only in a dimeric form, which would remain active since Asn631 at the A–D and B–E interfaces. Three glycosylation sites are present on the surface of the tetramer.

### Lysosomal Targeting Motif

The critical step in lysosomal targeting of soluble lysosomal enzymes is the recognition by a UDP-N-acetylglucosamine lysosomal enzyme N-acetylglucosamine-1-phosphotransferase [22]. In cathepsin D one structural motif and the N-linked glycan chains at Asn70 and Asn199 were found to be important for targeting of cathepsin D to the lysosome by phosphotransferase [44]. In human GUS, residues from 179 to 201 were predicted to be involved in lysosomal targeting due to their structural homology with a second lysosomal targeting loop of cathepsin D (265 to 292) [23], [41]. We have compared this motif in the newly refined structure with the earlier structure [25] and observed, that some of the loops are identical. However a dramatic change was observed in the conformation of Lys197 in the new structure (**Fig. 4A**). In the current structure, Lys197 is oriented towards the glycan on Asn173. The distance between the side chain of Lsy197 and terminal sugar of the glycan chain is only 5.0 Å, while Lys197 is 21 Å away from Asn173. Orientation of the corresponding residue Lys203 and N-linked oligosaccharide chains linked to Asn70is similar in the structure of cathepsin D [41],[45]. This analysis suggests that despite the remarkable differences in the overall structure of GUS and cathepsin D, a similarity in lysosomal targeting motif make both enzymes a substrate for phosphotransferase, which targets them to the lysosomes (**Fig. 4C**). On the other hand, corresponding loops in other lysosomal proteins, such as arylsulfatase A [46] and AGA [47] have more distinct conformations.

**Figure 5:**
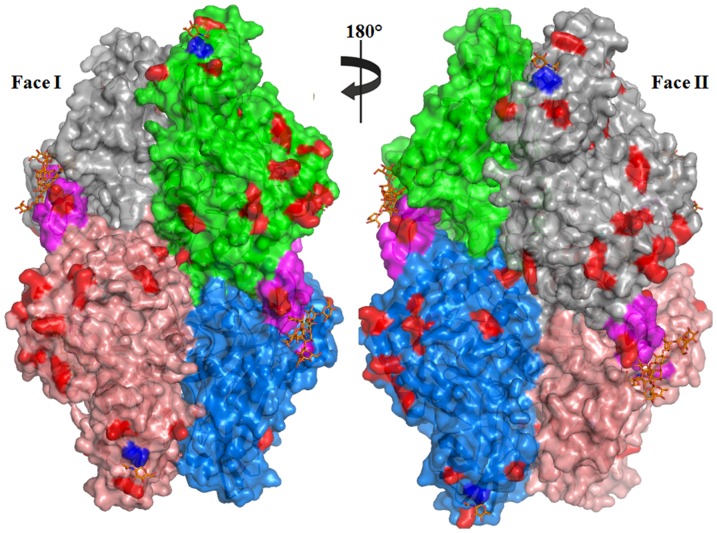
Surface representation of human GUS showing the surface lysines involved in phosphotransferase recognition. Residues of the lysosomal targeting loop and surface lysines are shown in pink and red respectively. N-linked glycan chains are shown in ball and stick model (orange) and Asn residues are colored in dark blue.

In AGA, site-directed mutagenesis studies suggested that phosphotransferase recognition may not involve a universal β-hairpin motif but be based on small contact points offered by lysine residues [29]. Critical roles for lysines in defining the recognition domain has also been supported by studies on cathepsin D and cathepsin L which showed significant decrease in mannose phosphorylation upon mutation of specific pairs of lysines in cathepsin D (Lys203 and Lys293) and cathepsin L (Lys54 and Lys99) [48]. Hence the lysine residues are themselves important for phosphotransferase recognition and binding to the enzyme and for phosphorylation of mannose residues by the catalytic subunit of the phosphotransferase [49]. It was also observed that these important lysines lie in close proximity to glycan chains. Similarly, we have observed that all four glycosylation sites have surface lysines in close proximity in human GUS. As shown in **Fig. 5** Asn173 has two neighboring lysines (Lys197 and Lys194). Similarly, Asn272 is in close proximity to Lys257 and Lys281, Asn402 to Lys333, Lys530 and Lys531, and Asn631 has neighboring lysines (Lys534 and Lys579). Cuozzoet.al., [48] proposed a model for the phosphorylation signal consisting of two lysine residues, exposed on the surface of the protein, which are spaced 34 Å apart and positioned in a specific orientation relative to the target oligosaccharide. This model was supported by studies on arylsulfatase A, where monoclonal antibodies against the epitope including the lysine cluster showed a dramatic inhibition of phosphotransferase recognition [50]. We have observed that Asn173 has two neighboring surface lysine residues (Lys197 and Lys194) situated at a distance of 22 Å and 27 Å, respectively. Similarly, Asn272 is 12 Å from two surface lysines (Lys268 and Lys281), and Asn631 is near to two surface lysines (Lys576 and Lys579) at a distance of 18 Å and 19 Å, respectively. The exception is Asn420 which does not have any neighboring surface lysine residues, but its glycan is Man6P based on other data.

### Comparisons with Bacterial GUS

**Figure 6:**
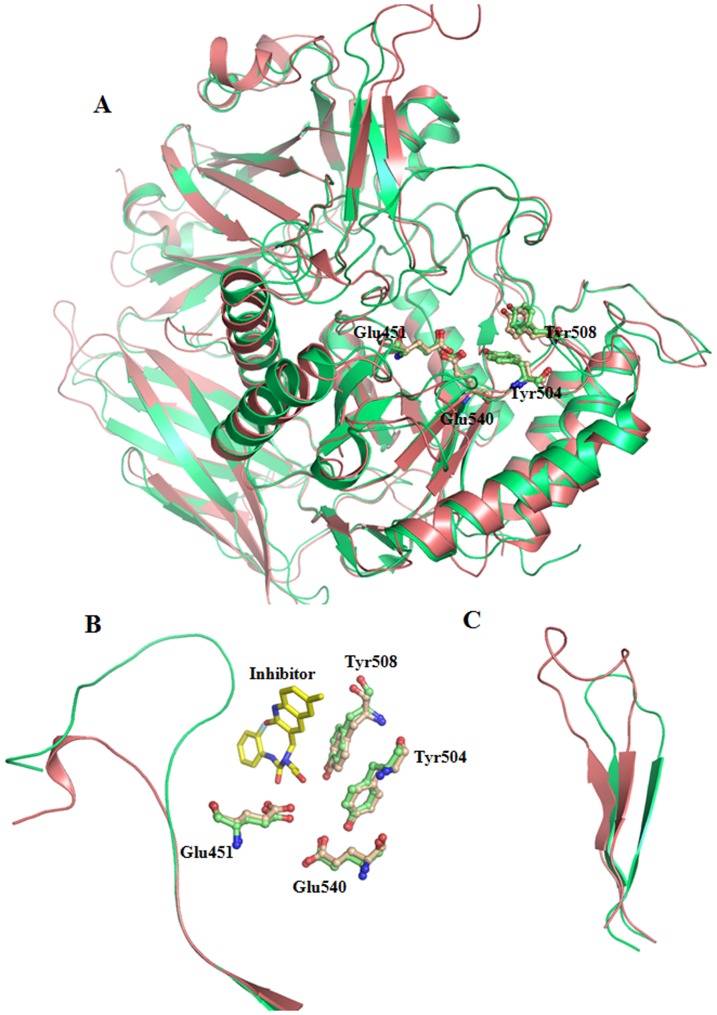
Structural comparison of human GUSB with bacterial GUS. (**A**). Cartoon model of superimposed human (light red) and bacterial GUS (green). Residues involved in catalysis are shown in ball and stick model for both the human (light red) and bacterial GUS (light green). (**B**). Superposition of the longer loop of bacterial GUS (light green) involved in binding to the inhibitor (yellow stick) and superimposed side chains of active site residues of human and bacterial GUS. (**C**). Comparison of lysosomal target loop of human GUS with bacterial GUS. Atomic coordinates of bacterial GUS were taken pdb code 3LPG [51].

Recently the crystal structure of bacterial GUS has been determined in the apo form and in complex with inhibitor [51]. We compared the refined structure of human GUS to bacterial GUS. Both structures are superimposable with an *r.m.s* deviation of 1.06 Å for C^α^ atoms [RCSB Protein Data Bank (pdb) codes: 3LPG with 3HN3], despite a relatively low sequence similarity (45%). Furthermore, the side chains of active site residues of both GUS enzymes are completely super imposable (**Fig. 6A**). However, a remarkable difference was observed in the loop (Ser360 to Glu378) near the catalytic site of bacterial GUS. Such a loop is completely absent in human GUS (**Fig. 6B**). This loop is formed by 17 extra residues in bacterial GUS as is evident from the sequence alignment (**Fig. 1**). Furthermore, this loop in bacterial GUS shows a close interaction with the inhibitor, (3-(2-fluorophenyl)-1-(2-hydroxyethyl)-1-[(6-methyl-2-oxo-1,2-dihydroquinolin-3-yl)methyl]urea)) at the entrance to the active site cavity, explaining its role in inhibition and catalysis, and making it a therapeutic target against bacterial GUS [51]. In the crystal structure of *E. coli* GUS in complex with potent inhibitors, 17 residues of the loop interact with inhibitor. Absence of these 17 residues in mammalian GUS explains why a potent inhibitor of bacterial GUS does not inhibit human GUS, despite the overall structural similarity of the enzymes.

Another difference is observed in the lysosomal targeting loop, which is much shorter in bacterial GUS. This difference is due to four extra residues (Gly198-Val201) which are not present in bacterial GUS. However, the neighboring residues are highly similar in both bacterial and human GUS, including critical Lys197 (**Fig. 6C**). Such an extension can be important for proper orientation of Lys197 as discussed above. It would be interesting to test the importance of these four residues to lysosomal targeting of GUS experimentally by deletion analysis.

### Comparisons with Related Proteins

Homologous structures of GUS were identified with DALI (www.ebi.ac.uk/dali). Human GUS shows close structural similarities with many proteins despite low sequence similarities (10-25%) (**Table 2**). Structurally, GUS is highly similar to β-galactosidase (Lac Z) [6], [52] and β-mannosidase [53]. Both human GUS and β-galactosidase enzymes possess a similar multi-domain structure including a jelly roll barrel, an immunoglobulin constant region domain, and a TIM barrel. The active sites of both of these proteins are structurally similar, a significant difference is that *E. coli* β-galactosidase is a metalloenzyme and requires Mg^2+^ for the catalysis whereas GUS has no such requirement [54].

**Table 2:** List of proteins showing structural similarities with human GUS.

S. No.	Pdb code	Protein Name	Source of Enzyme	z-score	aN- Align	bN-Res	cRMSD	Sequence Identity
1	3HN3	β-Glucuronidase	*Homo sapiens*	69.6	0.0	608	0.0	100
2	3K4D	β-Glucuronidase	*Escherichia coli*	49.0	572	597	1.3	46
3	1DP0	β-Galactosidase	*Escherichia coli*	33.6	515	1011	2.4	24
4	2VL4	β-Mannosidase	*Bacteroides thetaiotaomicron*	30.8	503	841	2.9	16
5	1RH9	Endo-β-Mannanase	*Solanum lycopersicum*	27.1	271	370	2.5	16
6	1UWI	β-Glycosidase	*Sulfolobus solfataricus*	24	257	489	2.4	15
7	1NP2	β-Glucosidase	*Thermusnon proteolyticus*	24	262	426	2.7	16
8	1DWJ	Myrosinase	*Sinapis alba*	22.9	260	499	2.8	14
9	1WCG	Thioglucosidase	*Brevicoryne brassicae*	22.9	259	462	2.8	11
10	1V03	Dhurinase	*Sorghum bicolor milo*	22.5	260	484	2.9	15
11	1ECE	Endocellulase E1	*Acidothermus cellulolyticus*	22.1	265	358	3.2	14
12	2OSX	Endoglycoceramidas	*Rhodococcus sp.*	20.4	255	449	2.9	15
13	1UR0	Galactanase	*Bacillus licheniformis*	20.1	256	386	3.3	14
14	3MMW	Endoglucanase	*Thermotoga maritima*	20.1	243	309	2.8	14
15	2VZO	Exo-β-D-Glucosaminidase	*Amycolatopsis orientalis*	19.5	499	850	3.1	15
16	2C8N	Alpha-L-Arabinofuranosidase	*Clostridium thermocellum*	19.5	241	497	2.8	12
17	2VZT	Exo-β-D-Glucosaminidase	*Amycolatopsis orientalis*	19.4	498	850	3.1	14
18	2JEP	Xyloglucanase	*Paenibacillus pabuli*	18.7	257	363	3.2	12
19	3NIY	Endo-1,4-β-Xylanase	*Thermotoga petrophila*	18.4	249	325	3.4	16
20	1PX8	Xylosidase	*Thermoanaerobacterium saccharolyticum*	17.6	249	500	3.2	12

a Number of residues aligned.

b Total number of residues.

c Root mean square deviations for C^α^ atoms.

**Figure 7:**
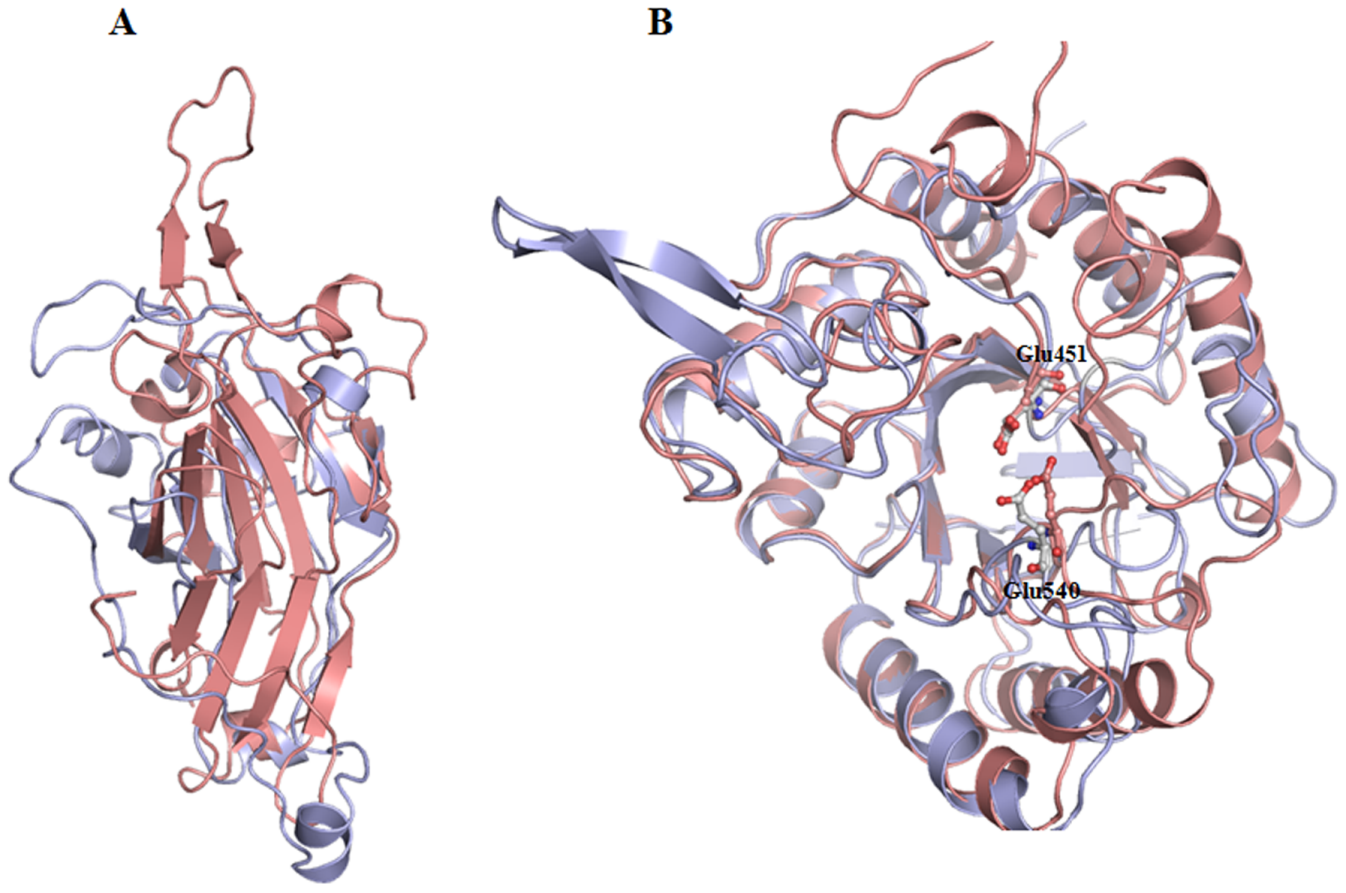
Cartoon model of superimposed human GUS and bacterial β-galactosidas. (**A**). Roll jelly like domain and (**B**). TIM barrel domains. Human GUS is shown in light red and bacterial β-galactosidase in sky blue. The side chains of active site residues of human and bacterial GUS are shown in ball and stick. Atomic coordinates of bacterial protein were taken from pdb code 1DP0 [52].

The jelly roll domain of GUS is superimposed to both β-galactosidase from *E. coli* and β-mannosidase from bacteroides with the *r.m.s* deviation for C^α^ carbon atoms of 1.8 Å and 2.1 Å, respectively (**Fig. 7A**). However, the GUS lysosomal targeting loop that is absent in both β-galactosidase and β-mannosidase proteins. A second immunoglobulin like domain of GUS is quite similar to that of corresponding domains in β-galactosidase and β-mannosidase (not shown). The third domain (TIM barrel) of human GUS is also comparable to that of β-galactosidase and β-mannosidase, and contains active site residues (**Fig.7B**). Interestingly, the side chains of active site residues are also identical in three-dimensional space. The TIM barrel domains are characteristic feature of many glycosyl hydrolases. Hence, this domain is structurally identical to many glycosyl hydrolases, despite limited sequence identities (**Table 2**). These findings suggest that all glycosyl hydrolases evolved from a common ancestor and acquired extra residues that confer substrate specificity and allow hydrolases to perform different functions in different sub-cellular localizations.

### Conclusions

We have refined the structure of human GUS at 1.7 Å resolution, and observed several new features in the structure including extra glycosylation, which was not seen in the earlier structure at 2.6 Å resolution. The three-dimensional structure of the lysosomal targeting loop was refined, adding to understanding of the structural basis of lysosomal targeting. Prior biochemical studies implicated that surface lysines are the key elements for phosphotransferase recognition of lysosomal enzymes. Correspondingly, multiple lysine residues are found in the vicinity of potential glycosylation sites of human GUS. The overall structure of human GUS is almost identical to that of bacterial GUS, except for the difference in the lysosome targeting and active site vicinity loops. The refined structure of human GUS showed close structural similarities with most of the glycosyl hydrolases. Our structure analysis, combined with an extensive list of mutations causing GUS deficiency in human and other site-directed mutagenesis studies provide a better understanding of the mechanisms of lysosomal targeting.

## Materials and Methods

### Protein Expression and Purification

Gene cloning and expression of human GUS was described earlier [32]. The highest-producing clone of a stably-transfected CHO cell line (CHO-K1), was scaled up, and secreted enzyme was collected for the purification of native GUS using an immunoaffinity chromatography procedure described in detail elsewhere [55]. Briefly, conditioned medium from CHO cells over-expressing GUS was centrifuged at 15,000 g for 30 minutes and an equal volume of 10 mM Tris (pH 7.5), 10 mM sodium phosphate, 0.5 M NaCl and 0.025% sodium azide was added. The clear medium was applied at a rate of 25 ml/h at 4°C to a 5-ml column of anti-human GUS Affigel 10 preequilibrated with the same buffer. The column was washed extensively with the same buffer to remove unbound proteins. Bound protein was eluted at with 3.5 M MgCl_2_ in 10 mM sodium phosphate (pH 5.0). Fractions containing GUS activity were pooled and desalted on a BioGel P-6 column preequilibrated with P6 buffer (25 mM Tris, pH 7.5, 1 mM β-glycerol phosphate, 0.15 mM NaCl, 0.025% sodium azide) to remove the MgCl_2_. The purified protein was dialyzed in 10 mM Tris pH 7.5 for further use.

### Crystallization

Freshly purified sample of protein was concentrated in buffer containing 50 mM Tris-HCl pH 7.5, with a final concentration of 2-3 mg/ml. Crystallization of GUS was performed by the vapor diffusion method in which an equal volume of protein was mixed with mother liquor containing 30% of MPD [56]. The best quality crystal was observed in 50mM Tris (pH 7.5) and 15% of MPD after three weeks at 25°C. Crystals were further improved by seeding and increasing the protein concentration up to 5 mg/ml.

### Data Collection and Processing

GUS crystals were cryoprotected by the addition of 10% glycerol, mounted on nylon loops and flash-frozen in liquid nitrogen at 100°K. Data were collected using the APS BEAMLINE 17-ID of synchrotron source, at a wavelength of λ = 0.98 Å on Bruker AXIOM 200 CCD detector. Data were processed with AUTOMAR and SCALEPACK from HKL package [57]. The overall completeness of the data was 88% at 1.7 Å resolution. The results of data collection are given in Table 1.

### Structure Determination and Refinement

The structures of human GUS were determined by a molecular replacement method using the coordinates of the earlier structure of human GUS at 2.6 Å resolution (pdb code: 2BHG) [25] with the CCP4 suite [58]. The molecular replacement solution was subjected to rigid-body refinement using the CNS program for whole molecule refinement [59]. The initial models were improved by repeated manual model buildings using the Coot program [60]. The structure was refined with the REFMAC 5.5 program [61]. The tight main-chain and side-chain non-crystallographic symmetry restraints between four crystallographically independent monomers A, B, D, and E, were used only in the initial refinement steps and were not used in final refinement cycles. The final model contains four monomer of GUS named as A, B, D and E, each having residues 22 to 631 and 2971 bound water molecules. The structure was refined to the R_cryst_ and R_free_ factors of 20.7% and 24.4%, respectively. The refined structure was submitted to the protein data bank (pdb code: 3HN3). Data collection and refinement parameters are shown in Table 1.
