# Correction: High Resolution Crystal Structure of Human β-Glucuronidase Reveals Structural Basis of Lysosome Targeting

**DOI:** 10.1371/journal.pone.0138401

**Published:** 2015-09-14

**Authors:** Md. Imtaiyaz Hassan, Abdul Waheed, Jeffery H. Grubb, Herbert E. Klei, Sergey Korolev, William S. Sly

Fig 1 is incorrect. Please see the corrected Fig 1 here.

**Fig 1:**
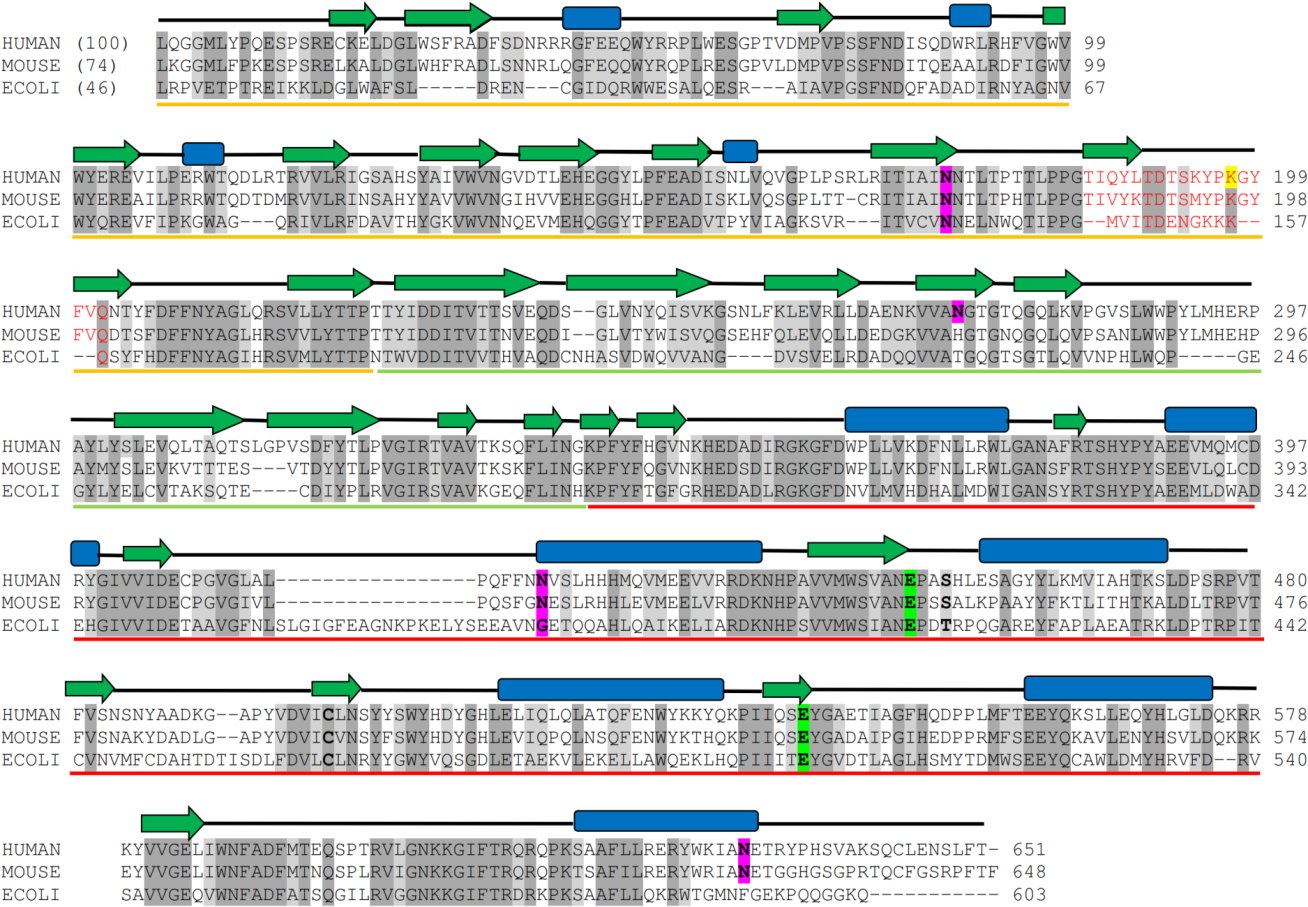
Multiple sequence alignment of human GUS with mouse and bacterial GUS. The percent sequence identities are given in parentheses. Completely conserved residues and homologous residues are shaded in dark and light grey, respectively. The secondary structure elements are given on the top of sequences, where α-helices are represented by blue rectangles, β-strands by green arrows. Domains 1, 2 and 3 are indicated by yellow, green and red line respectively, below the sequence. Conserved active site residues are highlighted in green boxes. Potential glycosylation sites are in pink. Glycosylation sites are in magenta boxes. Amino acid sequences of GUS were taken from the Uniprot database with their primary accession number as: human, P08236; mouse P12265; and E. coli, P05804.
